# Systems Biology Approaches Applied to Regenerative Medicine

**DOI:** 10.1007/s40139-015-0072-4

**Published:** 2015-02-10

**Authors:** Laura E. McNamara, Lesley-Anne Turner, Karl V. Burgess

**Affiliations:** 1 Centre for Cell Engineering, University of Glasgow, Glasgow, G12 8QQ UK; 2 Glasgow Polyomics, TCRC, University of Glasgow, Garscube Campus, Glasgow, G61 1QH UK

**Keywords:** Systems biology, Epigenomics, Transcriptomics, Proteomics, Metabolomics, Regenerative medicine

## Abstract

Systems biology is the creation of theoretical and mathematical models for the study of biological systems, as an engine for hypothesis generation and to provide context to experimental data. It is underpinned by the collection and analysis of complex datasets from different biological systems, including global gene, RNA, protein and metabolite profiles. Regenerative medicine seeks to replace or repair tissues with compromised function (for example, through injury, deficiency or pathology), in order to improve their functionality. In this paper, we will address the application of systems biology approaches to the study of regenerative medicine, with a particular focus on approaches to study modifications to the genome, transcripts and small RNAs, proteins and metabolites.

## Introduction

Regenerative medicine concerns the repair or replacement of damaged, diseased or otherwise poorly functioning tissues and organs, with the aim of restoring biological functionality. Systems biology offers many opportunities at the research and clinical level for the experimentation and facilitation of regenerative medicine, by providing models that predict the effects of drugs, diseases or treatments on biological systems at multiple levels and allow the contextualisation of changes, whether stoichiometrically or quantitatively in terms of the interacting networks of genes, transcripts, proteins, metabolites and phenotypic data. While a basic theoretical model of a biological system may be obtained by genome-wide reconstruction, to be relevant, it should be refined with recourse to high-density ‘omics approaches. There are specific advantages and disadvantages unique to each technique and –omic system of interest, but broadly the approaches offer the production of complex, in-depth, data-rich datasets that offer a snapshot insight into the epigenomic, transcriptomic, proteomic or metabolomic profile of the cells of interest at a particular point in time. This is an important point to note, as each of these systems is dynamic and can be modulated with time. Analysis at different time-points can offer some insight into the stability of particular results over time, and systems biology often necessitates that the data are obtained at intervals over a time series, in order to investigate the dynamics of the system of interest. Systems biology requires an iterative approach whereby the models can be created, tested, refined and re-tested, with the aim of ultimately producing models that approximate the reality of the biological systems of interest.

In this paper, we discuss various analytical techniques that can be used to investigate systems biology and studies that have begun to apply this to the study of regenerative medicine.

### Systems Biology: Modelling Biological Systems

Huang et al. elegantly discuss the properties of systems biology applied to stem cell biology. The authors describe the properties of the stem cell network in terms of its state space (the ‘three-dimensionality’ of large numbers of interconnected pathways and networks as they feed into and affect each other, in contrast to the more linear pathway descriptions), the high dimensionality of the system (comprising large numbers of variables) and the heterogeneity of the system (to account for the discrepancy between the implicitly assumed homogeneity of tissue cultures and the heterogeneity of actual populations) [1••].

**Fig. 1:**
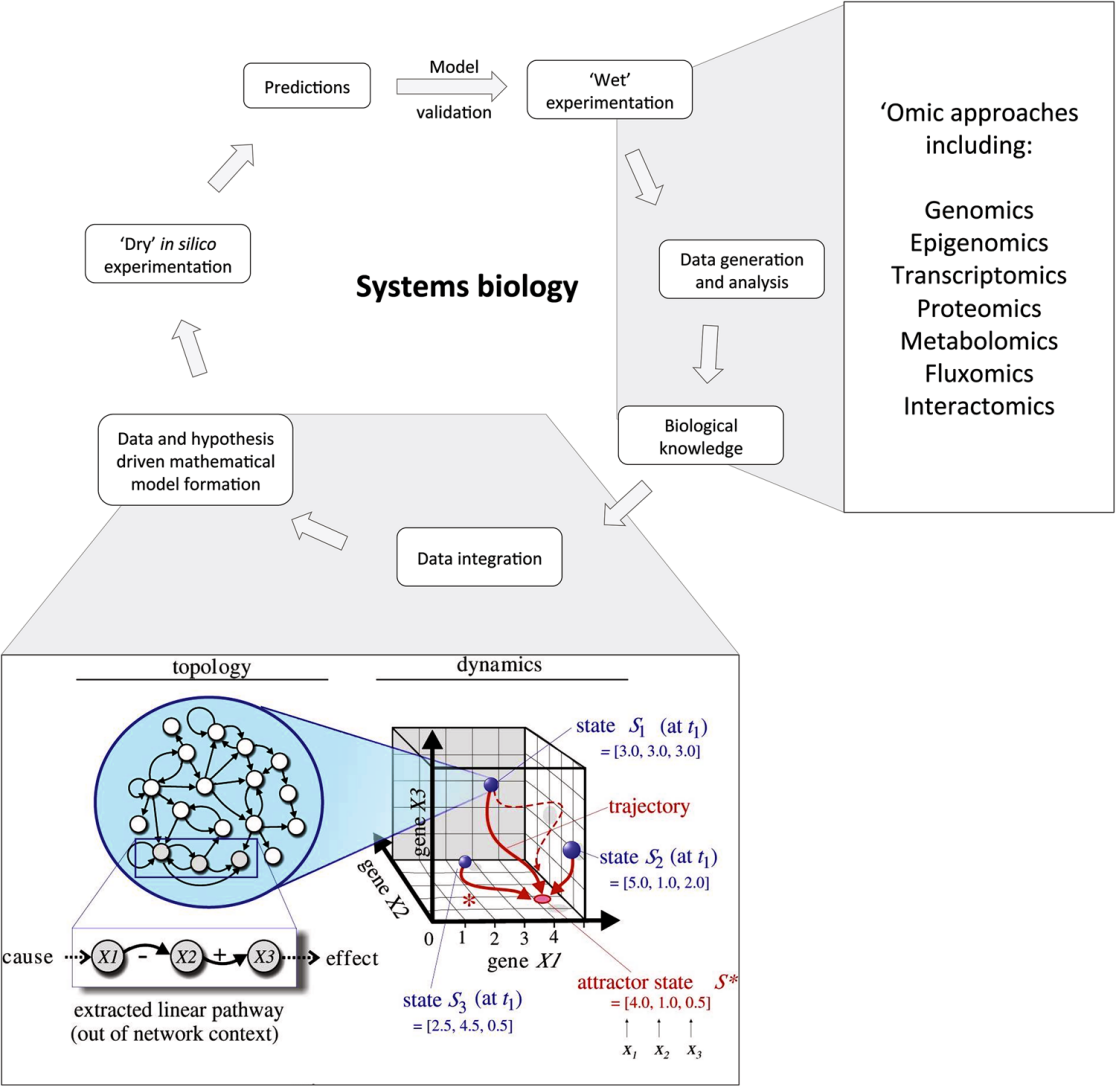
Schematic overview of the systems biology process, illustrating the iterative cycle between prediction and experiments and highlighting methods of data generation and integration of ‘wet’ experimental data (such as –omics data) for the refinement of the mathematical models.* Inset* image illustrates part of the modelling process, comparing the ‘topology’ of a gene network in terms of linear pathway delineation (a more traditional biochemistry approach) with its position within a network of interactions at a given point in time, and shows how this can be expanded into a three-dimensional dynamic state space model, where each of the topologies of the network exists at a particular state (*S*) at a particular point in time (*t*_1_), but that these are dynamic, and can tend towards a particular expression profile (or ‘attractor state’) if a suitable attractive state (such as a suitable stimulus to promote stem cell differentiation along a particular lineage) is present. *Inset image* (on topology and dynamics) reproduced from with permission from Huang [1••]

The networks have ‘traffic’ in different directions, based on the three-dimensionality of the integrated networks, which can be modulated with time, to accommodate the flux and directionality of movement in the system. This equates to network features such as gene expression levels, protein and metabolite abundance and post-translational modifications. These dynamic models begin to mathematically describe the interactions between different systems in order to approximate the reality of the systems. Steady-state models account for the equilibrium reached when the dynamic flux in the system tends to balance as a result of the multi-level interactions between different factors in the system. Certain nodes in the system account for preferred states (or ‘attractor’ states) that tend to have multiple routes that result in these outcomes, in order to buffer the system and stabilise it against disruptive influences. Critically, these mathematical models require the incorporation of real-life data to adjust and improve the fit of the models to the real-life situation. Figure 1 illustrates the interaction between the state space, the high dimensionality and heterogeneity and the requirement for the incorporation of ‘omics data into the models in order to improve the accuracy of the model and its capacity for effective prediction of particular outcomes given a particular set of parameters or modifiers to the system, such as prediction of the effect of a drug on the system, or the differentiation state of a stem cell.

### Epigenetic Modifications, Small RNA and Transcript Analysis

DNA is associated with histone proteins in structures called nucleosomes that can be moved by the DNA remodelling machinery, enabling conformational changes in the DNA architecture that modulate the accessibility of the gene promoters to the transcriptional machinery. Epigenetic histone modifications (or ‘marks’) are a ‘language’ of post-translational modifications (principally phosphorylation, acetylation, methylation and SUMOylation of specific amino acids, including lysine and serine) that can contribute to the repression, or facilitation, of gene expression by altering interactions with chromatin remodelers and affecting chromatin dynamics [2].

Epigenomics is the study of this process at the global level, including the histone modifications, methylation state of the DNA (which has roles in development and some pathologies) and other architectural changes to the chromatin. There is currently an EMBO-funded project underway (4DCellFate) which seeks to understand the interactions between different nucleosome proteins and remodellers across the stem cell genome. Such multi-level systems biology approaches offer the potential for a much more integrated vision of the pivotal fundamental processes in regenerative medicine, such as stem cell fate determination. Meissner et al. examined the methylation pattern in pluripotent stem cells and differentiated cells using a bisulphite sequencing approach, and found that the pattern of methylation of CpG islands (CG-rich sequences of DNA) was distinct between stem cells, stem cells under prolonged culture in vitro and differentiated cells, which has relevance to both fate determination and pathological states [3]. Epigenomics is discussed further in the context of pluripotent and induced pluripotent stem cells in [4].

To study gene transcripts at the global level, two main approaches are commonly used, namely next-generation sequencing (NGS) and microarrays. Microarrays are printed arrangements of oligonucleotide probes that enable the simultaneous comparative analysis of the abundance of thousands of transcripts. We have previously discussed the application of microarray analysis in the context of biomaterials samples, including technical details about the application of this approach, and thus this will be discussed in more brevity in the current review [5].

Microarrays are available for examining the abundance of transcripts and also of small untranslated RNAs. The examination of transcript abundance has the advantage that even low-abundance samples can be studied, as the RNA can be amplified once converted to complimentary DNA (cDNA). Some protocols, such as the 100 ng input modification for the Affymetrix HuGene 1.0/2.0 ST arrays, are particularly useful for minimising sample input. Interpretation of microarray data has the caveat that the transcripts are not necessarily being actively translated, although this can be circumvented using a modified technique called polysome analysis to harvest the fraction of mRNAs which are associated with the translational machinery (ribosomes). This issue does not apply to the study of small untranslated RNAs, as these RNA species are directly functional in RNA editing (e.g. small nucleolar RNAs, or snoRNAs) or repressing the translation of other transcripts (e.g. microRNAs, or miRNAs). Small RNA arrays are available which enable the analysis of thousands of small RNAs, but generally focus on miRNAs. Small RNAs have been shown to have importance in chromatin remodelling [6] and stem cell differentiation [7], and both snoRNA and miRNA species have been linked with the cellular response to topographically patterned surfaces, including the stem cell response to osteogenic topographies which could be used to pattern orthopaedic implants [8–10].

Systems biology approaches to transcriptomics seek to capture networks of correlated gene expression, in order to gain insight into the interaction between gene products. This includes large-scale, pair-wise comparisons of expressed genes, to examine which genes could be co-regulated, and the inter-relation of different datasets from different –omic approaches in order to refine and extend the theoretical and mathematical models that unite the datasets.

Although the large data yield is an advantage of systems biology, one of the challenges for the use of –omic techniques for systems biology is the vast quantity of complex data that results from it. Certain software programmes have been developed to aid visualisation and interpretation of results. The likely interactions between different differentially abundant RNA species can be mapped using software such as Ingenuity Pathways Analysis (http://www.ingenuity.com), which enables a useful overview of the interactions between different genes, and can also be utilised for the analysis of some other –omic techniques, including metabolomic data. This software also enables the prediction of likely phenotypic changes that would result from groups of key gene up- and down-regulations, for example, predictors of various stem cell fates. This was used recently to gather information about the predicted fate of stem cells cultured on osteogenic micro- and nano-topographically patterned titania substrates [9]. If certain genes are of particular interest, microarray analyses should ideally be validated using another technique, typically quantitative PCR (qPCR). A number of studies addressing aspects of regenerative medicine have also used other techniques to complement the results of microarray data, including immunohistochemistry, which was used by Hsiang et al. to examine the production of inflammatory cytokines and indicators of healing in a rat system using a model conduit [11].

NGS is a newer approach for the analysis of DNA (DNA-Seq), RNA (RNA-Seq) and chromatin immunoprecipitation (ChIP-Seq) samples. The deep sequencing approach adopted in this technique improves the accuracy of the sequencing data, as there should be multiple ‘reads’ of each sequence, which enhances the coverage in DNA-Seq. For RNA-Seq, deep sequencing has the advantage that the number of RNA species can be quantitated, which can give an estimate of gene expression levels, which is not available from microarray data [12]. Multiple samples can be run in a single lane, which enables efficient multiplexing, and in the example of RNA-Seq, the RNA is converted to cDNA (complementary DNA, which is the reverse complement of the RNA sequence), fragmented and adapters are added. Fragments are assembled into libraries and sequenced in short reads, which are assembled by comparison to a reference genome or sequences, or by assembling contiguous fragments in the absence of a reference. RNA-Seq is discussed further in [13, 14]. Rabani et al. used a combination of metabolic labelling and sequencing to study RNA dynamics (transcription, maturation and degradation) in murine cells, an approach which could offer valuable insight into the dynamics of RNA species in, for example, primary cells isolated from clinical samples, information which could help to bridge the gap between global transcript and protein data [15].

Compared with microarray analysis for transcriptomic studies, NGS has the advantage that previously uncharacterised RNA species can be detected in RNA-Seq, and the technique can also detect small RNA species (small RNA-Seq), including novel small RNAs. This technique has great potential for use in the field of regenerative medicine, since RNA-Seq can be used with scarce samples (down to ~10 pg in some cases) and can cope with some sample degradation, as the sequencing approach does not require polyadenylation of transcripts, which are useful for precious samples, such as clinical material.

Using a modified RNA-Seq approach termed ribosomal profiling, ribosomes can be isolated in order to identify the sections of mRNA which are ‘protected’ by the ribosomes to gain insight into the mRNA species being actively translated into the cell. Such analyses should help to improve the interconnection between different –omic datasets, such as transcriptomic and proteomic studies, which can be challenging to compare. In the context of regenerative medicine, these approaches would be valuable for evaluating, for example, gene expression in clinical samples using RNA-Seq, and promoter occupation by osteogenic transcription factors in stem cells exposed to different stimuli to correlate with gene expression patterns using ChIP-Seq. In addition, genome annotation provided by NGS can be cross-referenced with data produced by metabolomics and proteomics (techniques for the study of the global metabolite and protein profiles, as will be discussed) to gain insight into additional metabolic networks that had not been fully annotated. ChIP-chip is a term used to describe the combination of ChIP experiments and microarrays, to give a more comprehensive assessment of the binding sites in the genome, but unlike ChIP-Seq, this approach relies on known gene sequences being present in the array. The complexity of the data generated by NGS can present an issue, however, as vast datasets are generated, and the software pipelines available for the analysis and interpretation of these data are still maturing. Data analysis for DNA-Seq and RNA-Seq experiments is discussed further in [16].

### The Global Metabolite Profile

While analysis of the genome is now well established and proteome analysis continues to develop as a technology (now suitable for complete analysis of some moderate-sized proteomes) [17], the analysis of biological small molecules has lagged behind that of the other ‘omes’ until recently. The term ‘metabolome’ to describe the complete small molecule complement of a biological system was coined by Oliver in 1998 [18••], and the analysis of the metabolome became known as metabolomics or metabonomics.

Metabolites are the substrates and products of the chemical reactions that constitute life. As such, they comprise an enormously heterogeneous mixture of compounds and compound classes (e.g. sugars, amino acids, lipids, organic acids and biogenic amines). This alone leads to significant issues with the provision of metabolomics as a true ‘omic technology—methods ideal for the characterization of amino acids are likely to be poor for the characterization of steroids, for example. Despite this, most metabolomics platforms now detect hundreds to thousands of compounds, covering the majority of key biochemical pathways.

Careful design of experiments is absolutely critical to a successful metabolomic analysis. Broadly, an experiment will consist of control and treatment samples with replicate sets for each. Treatments may, of course, be series, such as time-courses, dose-curves or growth curves. The number of biological replicates is dictated by two factors: the variability of the metabolites in the sample, and the minimum fold-change expected to be detected in the samples. For example, clinical samples are likely to be extremely variable and often even small changes in metabolite concentration are of interest and thus hundreds to thousands of samples per state may be required for sufficient statistical power. In contrast, well-defined cell culture experiments (for example, where cells are exposed to drug treatments) may be characterized by low variability and the effects may be large, so a handful of replicates may be sufficient. We have previously discussed the application of metabolomics in the context of stem cell biology [19•], and in our experience, the use of a chloroform:methanol:water extraction buffer to directly harvest metabolites from cells adhering to biomaterial surfaces, such as titanium, has been valuable for the rapid quenching of metabolic reactions [19•, 20]. Quick quenching is essential for minimising ‘run-on’ metabolic reactions, and labile metabolites can be better retained by rapid sample processing. As with all –omic approaches, experimental consistency is important, as small changes in conditions, such as the volume of medium used in a cell culture experiment, or a change in the frequency of media changes, introduce unwanted additional variables and may impact upon the experimental results.

Metabolomics experiments may also be broadly characterized as ‘targeted’ or ‘untargeted’. In practice, all metabolomics experiments are in some way ‘targeted’ due to the inability of any single system to detect every metabolite in a system. There is a distinction, however, between ‘targeted’ methodologies [21, 22] that rely on detection of carefully defined panels of compounds, and ‘untargeted’ methods [23, 24••, 25] that use broad-based detection methods to detect as many features as possible. There are significant advantages to each method, but briefly, targeted methodologies generally provide more sensitive and rigorous quantitative data on a panel of detected compounds while having the limitation that one fails to detect compounds outside this panel. Untargeted methods detect many more compounds, but are more limited in terms of confidence of identification for the detected metabolites, and can suffer from reproducibility issues. The method selected depends significantly on the user’s knowledge of the system to be studied and their goals. For example, for hypothesis-driven analysis, such as researching energy metabolism [26], a targeted method may be most appropriate, however, if systemic effects are suspected, the pathway of effect is not known, or if it is suspected that an effect may occur at multiple foci, untargeted methodologies may be more appropriate.

Untargeted metabolomics can also be performed in a high-throughput manner to give greater insight into the dynamics of the metabolome of interest, as discussed in [27]. And although such approaches are currently generally restricted to the study of simple organisms such as bacteria, future adaptation of the approach for mammalian systems would have great potential for offering insight into the dynamics of metabolic changes in systems of interest in regenerative medicine. To better infer the functional relationships between different metabolites in mammalian cells, measurements of dynamic flux in the metabolome (known as fluxomics) can be made by rapid, repeated sampling. This flux can be modelled by ordinary differential equations (ODEs), as discussed in [28, 29]. Flux balance analysis uses steady-state models which make the assumption that there is no ongoing flux in metabolite concentration in the system, and make predictions based on the steady-state stoichiometry of the metabolites in the metabolic networks. A useful introduction to flux balance analysis is provided in [28, 30]. The isotope ^13^C can be used to examine the flux of metabolites, by adding labelled metabolites and tracking the proportions in which they are converted to other intermediates.

The two most commonly used methods of collecting metabolomic data are by mass spectrometry (MS) [23] and nuclear magnetic resonance [31]. MS has the advantage in sensitivity, while NMR has the benefits of higher reproducibility and absolute (rather than relative) quantitation. MS is commonly directly coupled to a separation system although direct infusion MS has been performed in large studies [32]. The most commonly used separation systems are liquid (LC) [33•] or gas [34] (GC) chromatography. GC is generally of much higher chromatographic resolution than LC, but LC has the benefit that no derivatisation of the sample is usually required (GC requires compounds that can be volatilized, so many small molecules must be chemically modified such that they vaporize on heating). An advantage of LC–MS is the enormous variety of separation chemistries that are available, allowing the researcher to cope with the diversity of metabolites. Reversed-phase chromatography is ideal for separating non-polar compounds such as lipids [24••], while HILIC chromatography is ideal for polar compounds such as amino acids [33•]. Ion chromatography may be used for ionic compounds such as organic acids [35]. Of course, no separation system is functional without a detector, and there is a huge diversity of MS equipment that may be coupled to chromatography systems. However, they may be broadly categorized as either useful for targeted analysis (Paul traps, single quadrupole and triple quadrupole instruments), or for untargeted analysis (time-of-flight instruments, orbitraps and Fourier-transform ion cyclotron resonance mass spectrometers). The latter instruments have undergone many developments in recent years to improve their capabilities for targeted analysis, but they still fall short of the sensitivity of a modern triple quadrupole instrument.

Whether one is performing a targeted or untargeted metabolomic analysis, the process of data analysis for MS is relatively unchanged. Data are collected in the form of proprietary raw data files and then processed to obtain defined peaks (whether detected de novo by an algorithm, or specified by the analyst at run time), which are collated across replicate sets and compared by their area or intensity to other replicate sets. Statistical analysis is performed and the dataset is put into context, either mechanistically, with reference to biochemical pathways, or in clinical studies, stratified with respect to clinical outcomes and patient data. Several open-source, cross-platform programmes that automate all or part of the process are available, such as MzMine [36], XCMS [37] and IDEOM [38]. When performing any analysis, it is critical to have a good understanding of the raw data, the statistics to be applied, the fundamentals of biochemistry and the biological question to be addressed.

We previously employed a metabolic approach to investigate the metabolic profile of stem cells cultured on osteogenic 15-nm-high titania nanopillars. This work highlighted the change from a metabolically quiet, quiescent skeletal stem cell profile on planar substrates to an ‘active’ metabolic profile on substrates inducing osteogenic differentiation. Specific metabolites involved in the osteogenic differentiation process were highlighted and of particular interest were a range of amino acids and sphingosine. This study agreed with other research that showed increased metabolic demand in cells undergoing osteogenesis in response to chemical stimulation [39].

In embryonic stem cells, plasticity was associated with an increased abundance of unsaturated (C=C double bonds) metabolites, which was reduced in differentiating cells [40•]. Weckwerth discussed the integration of –omic data from different sources, including NGS and metabolomic data [41].

### The Protein Dimension

Proteomics has utility in systems biology for its ability to capture information about post-translational modifications (such as phosphorylation and glycosylation), relative and absolute protein quantification and information to enhance interfacing and integration with other systems, since protein–DNA, RNA and protein–protein interactions can be detected using proteomic approaches.

DiGE (2D-fluorescence difference gel electrophoresis) is a technique for the two-dimensional resolution of protein abundance. In DiGE, proteins from control and test samples are labelled with different fluorescent CyDyes (typically Cy3 and Cy5), and in the first dimension, isoelectric focussing is performed to separate the labelled proteins by native charge, which utilises a high-voltage electric current to separate proteins along immobilised pH gradient (IPG) strips. In the second dimension, proteins are separated by molecular weight using the technique SDS-PAGE, to produce a ‘spot map’ of resolved proteins. The spot maps are scanned using a fluorescent scanner at suitable wavelengths to detect both fluorophores. Using comparative spot map analysis software such as DeCyder (GE Healthcare), the spot maps can be analysed to gain information about the relative abundance of various proteins between two or more conditions, and by comparison with a reference gel containing a higher protein load, this enables protein identification, by picking spots from the 2D gels, followed by MS. This was used to investigate protein expression levels in osteoprogenitors, stem cells and fibroblasts [42] cultured on micro- [42] and nanoscale [43] topographies.

Compared with traditional 2D gel electrophoresis, the technique has the advantages of increased sensitivity (due to the use of fluorescent CyDyes for protein labelling) and enhanced comparability between gels, as there is the option of including a pooled internal standard on each gel. Protein isoforms and post-translational modifications can also be detected using this approach. We and others have previously discussed experimental design for DiGE experiments [44•, 45•], technical adaptations of this technique for use with biomaterials samples [44•] and the modification of the technique for the detection and prevention of artefacts [46]. The use of saturation labelling (a type of CyDye labelling that aims to label all cysteine residues in the sample proteins with dyes with maleimide chemistry) greatly increases the sensitivity of the technique, which makes it particularly applicable to regenerative medicine, where clinical samples and other scarce or valuable protein sources are routinely used. Laser capture microdissection was used to examine protein samples from histological sections from clinical cancer samples, and this technique could be used to capture small amounts of tissue from retrieved implanted devices, organoids or similar scarce sources. In addition, as discussed in McNamara et al. [44•], sample pooling can be a valuable means of generating additional material, and the use of related protein sources (such as protein from tissue cultures, or comparable tissue close to an implant) can be useful in generating sufficient material for a reference gel when the sample source is low abundance. DiGE was used by Horrillo et al. to investigate the differentiation of mouse embryonic stem cells upon treatment with zebularine, an inhibitor of cytosine methylation [47]. DiGE was used to examine the proteome, in concert with microarray analysis to investigate the transcript profile of the cells, and epigenetic marks were examined using techniques including methylation-specific PCR, which specifically amplifies sites that have been methylated. These techniques were utilised alongside more traditional cell lineage evaluation approaches, such as immunostaining and flow cytometry. Fractionation enables the investigation of different sub-proteomes (such as nuclear, nucleolar or organellar compartments) by enriching for a particular subset of the proteome. Highly abundant proteins can also be depleted to enhance the detection of other less-abundant proteins, for example using albumin-depletion columns.

Some liquid chromatographic (LC)-based approaches, where LC separation is coupled to mass spectrometric protein identification, such as iTRAQ (isobaric tags for relative and absolute quantification), can be used to enable multiple comparisons between different conditions of interest and can be used to produce quantitative data on protein abundance. The ability to compare up to eight conditions against each other (in an ‘8-plex’ experiment) has great advantages for regenerative medicine, by enabling the comparison of the proteome under a variety of conditions, for example, following culture of cells on different biomaterials surfaces, or under different culture conditions. The iTRAQ approach was used to compare the protein profile of embryonic stem cells and the equivalent stem cells of teratocarcinomas [48].

Approaches such as stable isotopic labelling of amino acids in cell culture (SILAC) enable the comparative metabolic labelling of mammalian cells during culture, by incorporating heavy and light isotopes of amino acids during culture under two different culture conditions. This approach is useful for in vitro tissue culture experiments and, since the metabolic labelling is performed in living cells, avoids issues with labelling artefacts (which can occur with DiGE under some conditions) and changes to the proteome that can occur during protein extraction that could lead to the depletion of protein from one sample group, which can occur with post-extraction labelling approaches. Dynamic SILAC is useful for collecting information across a time series, enabling a systems biology approach to be adopted. Sobczyk et al. examined the effect of chemoattractants on the cytoskeleton of the slime mould *Dictyostelium* over a timescale of under a minute [49•]. Dynamic SILAC has also been used to investigate global protein turnover in human cells [50•], and pulsed SILAC was used to investigate modulation of the proteome in response to induction of miRNAs [51]. In the context of regenerative medicine, such techniques have the potential to offer valuable insight into the dynamics of the proteome under different conditions, for example, the protein signatures and biomarkers associated with cytocompatibility and immunotolerance of materials with time.

### Future Directions

In future studies, the incorporation of high-content imaging, such as immunofluorescence with microscopy suited to automated image collection, and analysis pipelines for the extraction of multidimensional image descriptors would be a valuable addition to systems biology studies in regenerative medicine. A high-content image approach was previously used to identify a ‘signature’ of cellular features that could be used for lineage prediction of stem cells at early time-points [52], and such a setup would have great potential for enhancing the predictive power of systems biology approaches.

## Conclusions

Systems biology aims to integrate data from a variety of global data sources, including genomics, fluxomics, metabolomics, proteomics, transcriptomics, epigenomics, DNA–protein interactions and interactomics. These techniques would benefit regenerative medicine as the data yield is high and increasingly comprehensive, and systems biology has the potential to gain more functional information from the datasets by modelling biological systems and enabling the prediction of states in response to defined changes to the system. This would be invaluable for modelling processes such as stem cell fate determination, predicting implant tolerance or reactivity and characterising the development of organoids.

## Abbreviations

ChIP: Chromatin immunoprecipitation
SDS-PAGE: Sodium dodecyl sulphate polyacrylamide gel electrophoresis
qPCR: Quantitative polymerase chain reaction

## References

[1] •• Huang S (2011) Systems biology of stem cells: three useful perspectives to help overcome the paradigm of linear pathways. Philos Trans R Soc B 366:2247–2259. *A very useful introduction to systems biology, which introduces the distinction between the more traditional biochemical pathways to the integrative systems approach, incorporating more facets of the system, including its dynamics, interactions, and the predictive element of computational modelling*10.1098/rstb.2011.0008 PMC3130416 21727130

[2] Strahl BD, Allis CD (2000). The language of covalent histone modifications. Nature.

[3] Meissner A (2008). Genome-scale DNA methylation maps of pluripotent and differentiated cells. Nature.

[4] Rada-Iglesias A, Wysocka J (2011). Epigenomics of human embryonic stem cells and induced pluripotent stem cells: insights into pluripotency and implications for disease. Genome Med.

[5] McNamara LE, Dalby MJ, Tsimbouri MP (2014). The use of microarrays and fluorescence in situ hybridization for the study of mechanotransduction from topography. Methods Cell Biol.

[6] Li L (2014). Chromatin remodeling by the small RNA machinery in mammalian cells. Epigenetics.

[7] Gangaraju VK, Lin H (2009). MicroRNAs: key regulators of stem cells. Mol Cell Biol.

[8] McMurray RJ (2011). Nanoscale surfaces for the long-term maintenance of mesenchymal stem cell phenotype and multipotency. Nat Mater.

[9] McNamara LE et al Osteogenic micro-nanopatterned titania for orthopaedic applications (manuscript in preparation)

[10] McNamara LE et al Nucleolar modulation and small RNA regulation by grooved topography (manuscript in preparation)

[11] Hsiang CY, Chen YS, Ho TY (2009). Nuclear factor-kappaB bioluminescence imaging-guided transcriptomic analysis for the assessment of host-biomaterial interaction in vivo. Biomaterials.

[12] Mortazavi A (2008). Mapping and quantifying mammalian transcriptomes by RNA-Seq. Nat Methods.

[13] Wang W, Gerstein M, Snyder M (2009). RNA-Seq: a revolutionary tool for transcriptomics. Nat Rev Genet.

[14] Ozsolak F, Milos PM (2011). RNA sequencing: advances, challenges and opportunities. Nat Rev Genet.

[15] Rabani M (2011). Metabolic labeling of RNA uncovers principles of RNA production and degradation dynamics in mammalian cells. Nat Biotechnol.

[16] Pepke S, Wold B, Mortazavi A (2009). Computation for ChIP-seq and RNA-seq studies. Nat Methods.

[17] Mann M (2013). The coming age of complete, accurate, and ubiquitous proteomes. Mol Cell.

[18] •• Oliver SG et al (1998) Systematic functional analysis of the yeast genome. Trends Biotechnol 16(9):373–378. *The first paper to coin the term ‘metabolome’. This review describes progress on the ‘omic analysis of the yeast phenotype*10.1016/s0167-7799(98)01214-1 9744112

[19] • McNamara LE et al (2012) Metabolomics: a valuable tool for stem cell monitoring in regenerative medicine. J R Soc Interface 9:1713–1724. *Discussion of non-dynamic metabolomic experiments in the context of stem cell biology, including an introduction to metabolomics, and considerations for experimental design and metabolite extraction for samples relevant to regenerative medicine*10.1098/rsif.2012.0169 PMC3385772 22628210

[20] McNamara L (2011). Skeletal stem cell physiology on functionally distinct titania nanotopographies. Biomaterials.

[21] Shlomi T (2014). Quantitation of cellular metabolic fluxes of methionine. Anal Chem.

[22] Botte CY (2013). Atypical lipid composition in the purified relict plastid (apicoplast) of malaria parasites. Proc Natl Acad Sci USA.

[23] Vincent IM (2014). Untargeted metabolomic analysis of miltefosine action in Leishmania infantum reveals changes to the internal lipid metabolism. Int J Parasitol Drugs Drug Resist.

[24] •• Dunn WB et al (2011) Procedures for large-scale metabolic profiling of serum and plasma using gas chromatography and liquid chromatography coupled to mass spectrometry. Nat Protoc 6(7):1060–1083. *This seminal paper describes in great detail the rigour that is involved in performing large-scale clinical metabolomic analysis, from sample preparation, through instrument performance monitoring and data analysis*10.1038/nprot.2011.335 21720319

[25] Sevin DC (2015). Biological insights through nontargeted metabolomics. Curr Opin Biotechnol.

[26] Selak MA (2005). Succinate links TCA cycle dysfunction to oncogenesis by inhibiting HIF-alpha prolyl hydroxylase. Cancer Cell.

[27] Fuhrer T, Zamboni N (2015). High-throughput discovery metabolomics. Curr Opin Biotechnol.

[28] Raman K, Chandra N (2008). Flux balance analysis of biological systems: applications and challenges. Brief Bioinform.

[29] Link H, Christodoulou D, Sauer U (2014). Advancing metabolic models with kinetic information. Curr Opin Biotechnol.

[30] Orth JD, Thiele I, Palsson BO (2010). What is flux balance analysis?. Nat Biotechnol.

[31] Peyraud R (2009). Demonstration of the ethylmalonyl-CoA pathway by using 13C metabolomics. Proc Natl Acad Sci USA.

[32] Castrillo JI (2003). An optimized protocol for metabolome analysis in yeast using direct infusion electrospray mass spectrometry. Phytochemistry.

[33] • Kamleh A et al (2008) Metabolomic profiling using Orbitrap Fourier transform mass spectrometry with hydrophilic interaction chromatography: a method with wide applicability to analysis of biomolecules. Rapid Commun Mass Spectrom 22(12):1912–1918. *This paper describes the method for analysis of small polar metabolites that is becoming the standard in metabolomic analysis*10.1002/rcm.3564 18470888

[34] Fiehn O (2000). Metabolite profiling for plant functional genomics. Nat Biotechnol.

[35] Burgess K (2011). Semi-targeted analysis of metabolites using capillary-flow ion chromatography coupled to high-resolution mass spectrometry. Rapid Commun Mass Spectrom.

[36] Katajamaa M, Miettinen J, Oresic M (2006). MZmine: toolbox for processing and visualization of mass spectrometry based molecular profile data. Bioinformatics.

[37] Smith CA (2006). XCMS: processing mass spectrometry data for metabolite profiling using nonlinear peak alignment, matching, and identification. Anal Chem.

[38] Creek DJ (2012). IDEOM: an Excel interface for analysis of LC-MS-based metabolomics data. Bioinformatics.

[39] Reyes JMG (2006). Metabolic changes in mesenchymal stem cells in osteogenic medium measured by autofluorescence spectroscopy. Stem Cells.

[40] • Yanes O et al (2010) Metabolic oxidation regulates embryonic stem cell differentiation. Nat Chem Biol 6(6):411–417. *An exciting examination of the distinct metabolic profile of embryonic stem cells and differentiating cells*10.1038/nchembio.364 PMC2873061 20436487

[41] Weckwerth W (2011). Unpredictability of metabolism—the key role of metabolomics science in combination with next-generation genome sequencing. Anal Bioanal Chem.

[42] McNamara LE (2012). The role of microtopography in cellular mechanotransduction. Biomaterials.

[43] Kantawong FA (2009). Differential in-gel electrophoresis (DIGE) analysis of human bone marrow osteoprogenitor cell contact guidance. Acta Biomater.

[44] • McNamara LE et al (2009) Fluorescence two-dimensional difference gel electrophoresis for biomaterial applications. J R Soc Interface. doi:10.1098/rsif.2009.0177.focus. *Discussion of the use of 2D-DiGE in the context of regenerative medicine, with considerations of sample preparation and experimental design for the use of scarce samples*10.1098/rsif.2009.0177.focus PMC2843984 19570793

[45] • Tannu NS, Hemby SE (2006) Two-dimensional fluorescence difference gel electrophoresis for comparative proteomics profiling. Nat Protoc 1:1732–1742. *An in-depth protocol for the application of 2D-DiGE*10.1038/nprot.2006.256 PMC2001252 17487156

[46] McNamara LE (2011). Preventing and troubleshooting artefacts in saturation labelled fluorescence 2-D difference gel electrophoresis (saturation DiGE). Proteomics.

[47] Horrillo A (2013). Zebularine regulates early stages of mESC differentiation: effect of cardiac commitment. Cell Death Dis.

[48] Chaerkady R (2010). Comparative proteomics of human embryonic stem cells and embryonal carcinoma cells. Proteomics.

[49] • Sobczyk GJ, Wang J, Weijer CJ (2014) SILAC-based proteomic quantification of chemoattractant-induced cytoskeleton dynamics on a second to minute timescale. Nat Commun 5:3319. *An elegant examination of the potential for the study of rapid dynamic proteomic analyses*10.1038/ncomms4319 PMC3971484 24569529

[50] • Doherty MK et al (2008) Turnover of the human proteome: determination of protein intracellular stability by dynamic SILAC. J Proteome Res 8:104–112. *An interesting study of proteome-wide protein turnover using a dynamic SILAC approach with human cells*10.1021/pr800641v 18954100

[51] Selbach M (2008). Widespread changes in protein synthesis induced by microRNAs. Nature.

[52] Treiser MD (2010). Cytoskeleton-based forecasting of stem cell lineage fates. PNAS.

